# The ICP22 protein selectively modifies the transcription of different kinetic classes of pseudorabies virus genes

**DOI:** 10.1186/1471-2199-14-2

**Published:** 2013-01-29

**Authors:** Irma F Takács, Dóra Tombácz, Beáta Berta, István Prazsák, Nándor Póka, Zsolt Boldogkői

**Affiliations:** 1 Department of Medical Biology, Faculty of Medicine, University of Szeged, Somogyi B. st. 4, Szeged, H-6720, Hungary

**Keywords:** Herpesvirus, Pseudorabies virus, Real-time PCR, ICP22, *us1* gene

## Abstract

**Background:**

Pseudorabies virus (PRV), an alpha-herpesvirus of swine, is a widely used model organism in investigations of the molecular pathomechanisms of the herpesviruses. This work is the continuation of our earlier studies, in which we investigated the effect of the abrogation of gene function on the viral transcriptome by knocking out PRV genes playing roles in the coordination of global gene expression of the virus. In this study, we deleted the *us1* gene encoding the ICP22, an important viral regulatory protein, and analyzed the changes in the expression of other PRV genes.

**Results:**

A multi-timepoint real-time RT-PCR technique was applied to evaluate the impact of deletion of the PRV *us1* gene on the overall transcription kinetics of viral genes. The mutation proved to exert a differential effect on the distinct kinetic classes of PRV genes at the various stages of lytic infection. In the *us1* gene-deleted virus, all the kinetic classes of the genes were significantly down-regulated in the first hour of infection. After 2 to 6 h of infection, the late genes were severely suppressed, whereas the early genes were unaffected. In the late stage of infection, the early genes were selectively up-regulated. In the mutant virus, the transcription of the *ie180* gene, the major coordinator of PRV gene expression, correlated closely with the transcription of other viral genes, a situation which was not found in the wild-type (*wt*) virus. A 4-h delay was observed in the commencement of DNA replication in the mutant virus as compared with the *wt* virus. The rate of transcription from a gene normalized to the relative copy number of the viral genome was observed to decline drastically following the initiation of DNA replication in both the *wt* and mutant backgrounds. Finally, the switch between the expressions of the early and late genes was demonstrated not to be controlled by DNA replication, as is widely believed, since the switch preceded the DNA replication.

**Conclusions:**

Our results show a strong dependence of PRV gene expression on the presence of functional *us1* gene. ICP22 is shown to exert a differential effect on the distinct kinetic classes of PRV genes and to disrupt the close correlation between the transcription kinetics of *ie180* and other PRV transcripts. Furthermore, DNA replication exerts a severe constraint on the viral transcription.

## Background

The pseudorabies virus (PRV), an alpha-herpesvirus, is the etiological cause of Aujeszky’s disease of swine [1]. PRV is related to the human pathogen varicella-zoster virus (VZV) and herpes simplex virus types 1 and 2 (HSV-1 and -2), and the animal herpesvirus bovine herpesvirus type 1 (BHV-1). PRV is widely used as a model organism in investigations of the molecular pathomechanisms of the herpesviruses [2], and is a useful tool for the mapping of neural circuits [3,4]. Attempts have additionally been made to utilize this virus as a gene delivery vector [5,6] and an oncolytic agent [7]. Besides the lytic phase, alpha-herpesviruses can enter a latent state, where they transcribe a limited set of *cis*-antisense RNAs [8]. Traditionally, the lytic herpesvirus genes are classified into three kinetic categories: immediate-early (IE) genes, early (E) genes and late (L) genes. On a finer scale, an intermediate category, the early/late (E/L = delayed early) genes can also be distinguished [9]. PRV encodes a single IE gene, the *ie180* gene. The IE180 protein, a transactivator, is the principal coordinator of the overall gene expression of the virus. E genes encode proteins required for the nucleotide metabolism and DNA replication. Other E genes such, as the early protein 0 (*ep0*) [10] and *ul54* genes [11], encode transcriptional regulators. Most of the L genes code for structural elements of the virus. ICP22 is one of the five IE proteins of HSV-1, which is encoded by the *us1* gene. Intriguingly, a large part of the HSV *us1* gene is located in the unique US region, whereas its promoter and a short 5’ portion of the transcribed region are in the inverted repeat (IR) segment. In PRV, however, the entire *us1* gene (earlier called the *rsp40* gene) resides in the IR region; this gene is therefore represented in two copies in the PRV genome. There is no consensus as to whether the PRV *us1* gene is expressed in IE [12] or E kinetics [13]. We demonstrated in an earlier analysis that this gene is expressed in atypical kinetics, and that it is obviously not an IE gene [9]. The function of the ICP22 polypeptide has primarily been analyzed in HSV-1. The investigations have revealed that ICP22 is a multifunctional protein that plays roles in various aspects of HSV pathogenesis. It has not yet clearly established whether ICP22 acts to repress E genes [14] or to enhance the transcription of L genes [15]. It has been shown that not all L genes require this transactivator for their expressions [16]. The BICP22 protein of BHV-1, a homologue of ICP22, has been demonstrated to exert a general repressive effect on each kinetic class [17]. Rice and coworkers reported that ICP22 acts at the level of transcriptional regulation [18]. However, the level of ICP0 mRNA was also reduced in the *us1* knockout (KO) HSV [16], which raises the question of whether the direct cause of the reduced transcription is the lack of *us1* gene activity or the low ICP0 mRNA level. ICP22 has to be phosphorylated by the viral UL13 protein kinase in order to accomplish the transcriptional activation of L genes [19]. An additional function of the ICP22 polypeptide is associated with the alteration of the activity of cyclin-dependent kinase cdc2, a regulator of the cell cycle, which results in a selective up-regulation of HSV L genes during lytic infection [20]. Furthermore, HSV ICP22 also acts to modify the phosphorylation of RNA polymerase II (RNAP II) [21], which carries out the transcription of viral genes. One of the major control regions of RNAP II is its carboxy terminal domain (CTD), residing on the large subunit of the molecule. The CTD, containing multiple repeats of a heptapeptide sequence, serves as a binding site for various cellular proteins involved in the regulation of transcription. ICP22 is presumed to trigger the loss of Ser-2 phosphorylation on the CTD, and thereby modify the activity of RNAP II [22]. A novel function of ICP22 was recently identified, involving alteration of the chaperon localization of the host cells [23]. It has been shown that ORF63, the ICP22 homolog of VZV, does not alter RNAP II phosphorylation and the host chaperon machinery [22], which might indicate that ICP22 acts in a species- or genus-specific manner. In the present study, we have investigated the effects of *us1* gene deletion on the overall transcription of PRV genes.

## Results and discussion

### Experimental design

An insertion mutant PRV strain was constructed which contains the mutation in both copies of the *us1* gene. Mutation of a DNA sequence in the internal repeat region is copied to the terminal repeat by a mechanism called equalization [24]. From among the 70 PRV genes, 32 were selected for the transcription analysis, which reside at the upstream position of the tandem gene clusters and which represent each kinetic class of PRV genes. The reason for this choice was to exclude the distorting effect of the transcriptional read-through exerted by the upstream genes on the downstream genes. Furthermore, the genes selected for analysis play important roles in the regulation of the overall gene expression of the virus, including the *ie180*, the *ep0* (and their antisense transcripts, the LAT and the AST, respectively), the virion host shut-off (*vhs*), and the *ul54* genes. For each viral gene, a minimum of 3 parallel replicates were performed in order to achieve statistical reliability. Immortalized PK-15 cells were infected with either the wild-type (*wt*) or *us1* gene-deleted (*us1-KO*) PRV, using a high multiplicity of infection [MOI; 10 plaque-forming units (pfu)/cell]. The low-dose infections produce a much finer resolution of the cycle threshold (Ct) values for the transcripts than in the case of high-dose infections; however, in the former case a large proportion of the cells remain uninfected, which allows the initiation of an additional infection cycle after 6 h post-infection (pi) by the newly generated virions [9], which would confuse the interpretation of the expression data obtained. The transcription of PRV genes was monitored at 9 different time points: 0.5, 1, 2, 4, 6, 8, 12, 18 and 24 h pi (multi-timepoint analysis). Strand-specific primers were used for the reverse transcription reaction so as to exclude the distorting effects of the potential *cis*-antisense transcripts that might be produced from the complementary DNA strands, which cannot be avoided on the use of other methods, such as oligo-dT- or random priming-based reverse transcription [25]. On the other hand, we found that the specificity of strand-specific primer-based RT is much higher than that of other methods [9]. In our work, we applied a modified version of the mathematical model described by Soong and colleagues for the relative quantification [26]. Specifically, we used the average of the 6-h ECt-sample values of the *wt* PRV for each gene in both the *wt* and the mutant backgrounds, as controls, which were normalized to the average of the corresponding 28S values (ECt-reference). The 28S RNA gene was used as a reference gene since the ribosomal RNAs are not substrates of VHS ribonuclease [27]. We used a selected Ct value (at 6 h in our system) as control for the comparability of the relative copy numbers of a transcript at different time points. The relative amounts of the transcripts of different genes cannot be compared directly due to the variation in the primer efficiencies in both RT and PCR. However, the use of multi-timepoint qPCR analysis allows a comparison of the transcription kinetics of different viral genes throughout the whole period of infection. Furthermore, this method allows a comparison of the same genes in the two genetic backgrounds (R_r_ = R_us1KO),/_R_wt_), and of the mRNAs and the complementary antisense transcripts in the case of the *ep0*/LAT and *ie180*/AST pairs. A high R_r_ value indicates an inhibitory effect of the ICP22 protein on the transcript level of a particular gene in the *wt* virus. Conversely, a low R_r_ value indicates a stimulatory effect on the gene expression. We applied the same logic for the interpretation of the data obtained as is used in other knockout organisms, i.e. the normal role of the *us1* gene is considered to be the opposite of that of the phenotype caused by the mutation of this gene. In other words, an elevated expression of a gene in the *us1*-null mutant is indicative of a suppressive effect of the ICP22 protein on the expression of this gene.

### Confirmation of the mutant genotype

The mutation in the *us1* gene was confirmed by PCR amplification of the DNA sequences containing the mutation, followed by pyrosequencing. The mutation of the *us1* gene was rescued, and this was followed by growth analysis in order to confirm that the altered kinetic properties can be solely explained by the abrogation of the *us1* function (data not shown). Besides these analyses, we also carried out more precise techniques for this purpose. Thus, we compared the rates of increase of viral DNA during the first twelve hours of viral infection. We observed similar dynamics in the growth rates of the DNA of the wild-type and rescued viruses, while both differed significantly from those of the us1-mutant virus (Additional file 1). In addition, we compared the transcription kinetics of the two most important transactivator genes, the ie180 and ep0 genes of pseudorabies virus. This revealed that the kinetics of the rescued virus resembled that of the wild-type virus, but differed significantly from that of the mutant virus (Additional file 2 and Additional file 3).

### Transcription of the *us1* gene in the wild-type PRV

**Figure 1:**
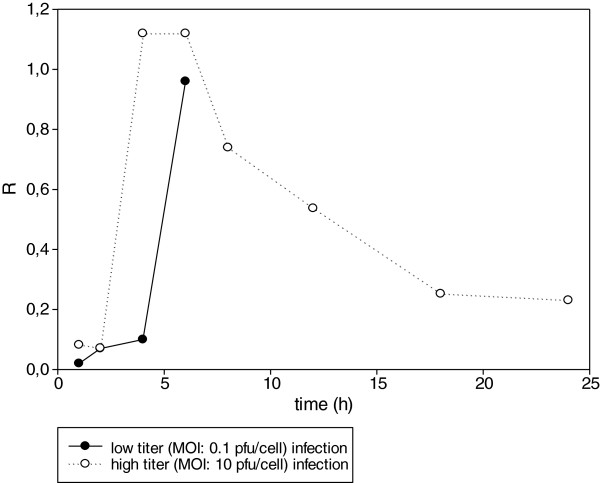
**Transcription kinetics of the *us1 *gene of the *wt *virus in low-MOI (0.1 pfu/cell) and high-MOI (10 pfu/cell) infection conditions.** The low-MOI experiment was conducted only up to 6 h pi: after this time, the newly generated virions may be released from the infected cells and initiate new infections in the originally non-infected cells. The Figure shows a 2-h delay in the initiation of the steep rise in gene expression.

We compared the expression kinetics of the *us1* gene of the *wt* virus under the low- and high-dose infection conditions (Figure 1). Infection was analyzed for 6 h in the low-MOI infection, and for 24 h in the high-MOI infection. The data revealed that in the low-MOI infection the *us1* gene behaved as an L gene [9], since the amounts of its transcripts started to rise rapidly from 4 h pi. This is in contrast with the situation of the high-dose experiment where this gene exhibited typical E characteristics in the early phase of infection, since its expression began to rise steeply from 2 h pi. Thus, there was a 2-h shift in the expression kinetics between the two experimental conditions up to 6 h pi. Another unusual feature of the *us1* mRNA kinetics was the rapid drop in the transcript level after 6 h pi, which is not typical in any of the kinetic classes of the viral genes.

### The impact of the *us1* gene mutation on the viral transcriptome

**Figure 2:**
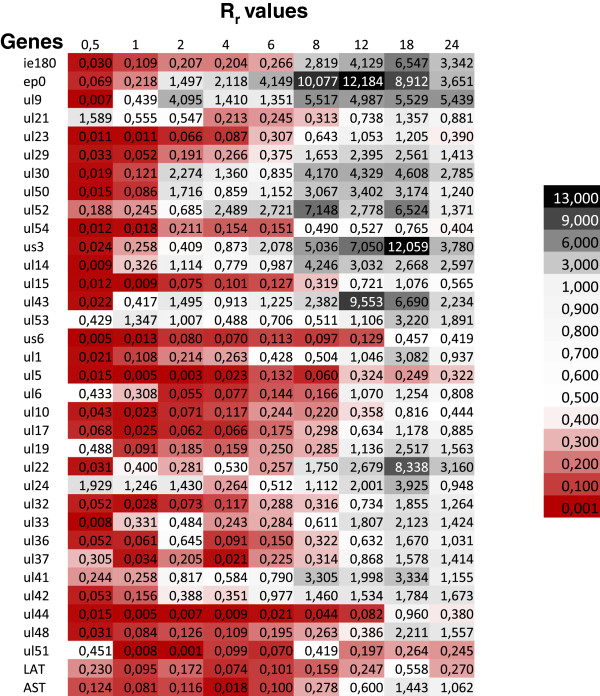
**Heatmap visualization of the ratios of the transcripts of the *us1-KO *and the *wt *viruses (R**_**r**_ **= R**_**us1KO**_**/R**_**wt**_**).** The red color (R_r_ < 1) indicates a transcript level that is lower in the mutant than in the *wt* virus at a certain period of infection, whereas the black (R_r_ > 1) indicates the opposite: the mutant virus produces a higher amount of mRNA than the *wt* virus.

**Figure 3:**
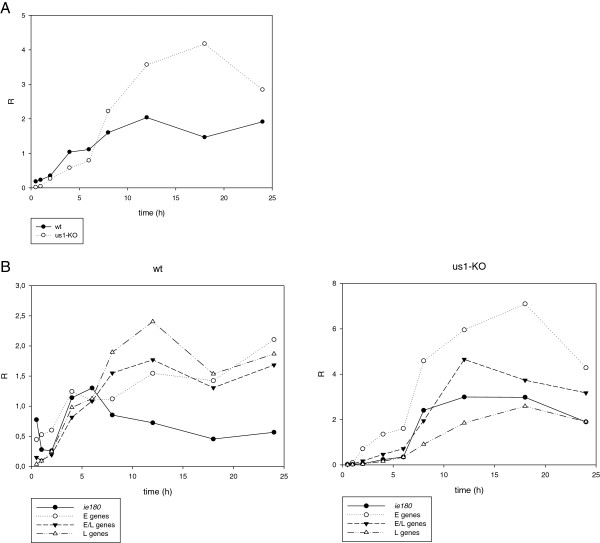
**The average relative expression ratios (**$\bar{R}$**). (A)** The $\bar{R}$ values of the total genes of the mutant and *wt* viruses plotted against time. The average level of the transcripts of the mutant virus is lower in the early stage, but higher in the late stage of infection. **(B)** The $\bar{R}$ values of the different kinetic classes of mutant and *wt* viruses. In the mutant virus, the L genes are repressed in the early stage of infection, whereas the E gene expressions are enhanced in the late stage of infection.

The R values of individual genes were calculated for both *wt* and *us1-KO* mutant viruses, and compared by calculation of the ratios (R_r_ = R_us1KO_/R_wt_) for each time point (Figure 2). The average R values were calculated and plotted for the total genes (

$$\bar{R}$$)

(Figure 3A; Additional file 4A) and for each kinetic class of genes (

$${\bar{R}}_{E}$$,

$${\bar{R}}_{\;E/L}$$

and

$${\bar{R}}_{L}$$.)

in both genetic backgrounds (Figure 3B; Additional file 4B). Additionally, the mutant and *wt* viruses were compared by calculating the average R_r_ values for the total genes (

$${\bar{R}}_{r}$$)

and separately for each kinetic class (

$${\bar{R}}_{\;r-E},R_{r-E/L}$$

and

$${\bar{R}}_{\;r-L}$$)

(Figure 4B). Figures 3A and 4 reveal a significant decrease in the amounts of transcripts in each kinetic class of genes in the first hour of infection (Figure 2). The average levels of transcripts were not decreased significantly or at all in a few genes (e.g. *ul21*, *ul24*, *ul53* and *ul19*), but in most genes, and especially the E genes, the fold of the decrease was close to 2 orders of magnitude at this very early stage of infection. However, the average level of the E transcripts of the mutant virus reached approximately the same level as that of the *wt* virus in the interval 1–6 h pi. This was in contrast with the L genes, which on average were still expressed at a significantly lower level during the first 8 h of infection. The average amounts of the E/L transcripts falls between those of the E and L genes. The *ie180* gene was also expressed at a lower level in the first 6 h of infection in the mutant virus. The deletion of the *us1* gene led to a significant reduction in the average expression of the L genes and to a lesser reduction in the expression of the E/L genes in the first 6 h pi, but the E genes were expressed at a slightly higher level in the mutant than in the *wt* background in the period 2–6 h pi (Additional file 4B). The average rate of transcription of the IE, E and E/L genes from the mutant viral genome exceeded the rate of transcription from the *wt* genome after 6 h pi (Figure 4). This was in contrast with the transcript levels of the average L genes of the mutant virus, which remained below or equal to that of the *wt* virus, except at 18 h pi. These data suggest that the ICP22 protein exerts an inhibitory effect especially on the transcription of the *ie180* and E genes following the initiation of viral DNA synthesis. The *us1* gene deletion exerted the highest impact on the expression of the *ep0* gene, an important regulator of PRV gene expression [28]: the amount of *ep0* transcripts was ~ 10 times higher in the cells infected with the mutant than that for the *wt* virus within the period 8–18 h pi. This result was the opposite of that described in the HSV, where the level of ICP0 mRNA was significantly lower in the KO virus [16]. The expressions of the *us3*, *ul22*, *ul43*, and *ul52* genes were also significantly elevated at certain time points in the late stage of infection (Figure 2). The *ul5*, *ul51* and LAT genes were the only examined PRV genes whose expressions were always lower in the *us1-KO* virus than in the *wt* virus. Overall, the above data suggest that the ICP22 protein exerts a selective effect on the expressions of PRV genes belonging in different kinetic classes. ICP22 (possibly of tegument origin) appeared to have a significant stimulatory effect on the general gene expression of the virus at both 30 min and 1 h pi [PRV ICP22 has been shown to be localized in the viral tegument layer [29]. Though to a lower extent, its stimulatory effect continued up to 6 h in the E/L and L genes, but the effect became neutral or slightly inhibitory in the later stages of infection in these kinetic classes of genes. The expressions of the E genes exhibited different profiles on average in the mutant background: no discernible effect of the mutation within the 1–6 h pi period, followed by a profound inhibitory effect in the later phases of infection. Our results may resolve the debate as to whether ICP22 represses the E genes [14] or enhances the L genes [15]. The data demonstrate that the rates of transcription of the L genes in the mutant virus are repressed (and therefore they are selectively enhanced in the *wt* virus) in the very early phase of infection, whereas the rates of transcription of the E genes are selectively enhanced (and therefore they are repressed in the *wt* virus) in the late phase of infection.

**Figure 4:**
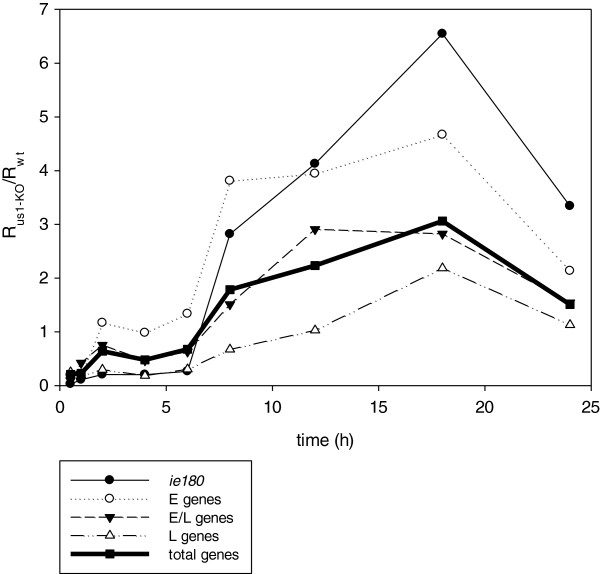
**The average R**_**r**_**values (**${\bar{R}}_{r}={\bar{R}}_{\;\text{us}1-\text{KO}}/{\bar{R}}_{\;\text{wt}}$**).** The thick solid line depicts the ${\bar{R}}_{r}$ values of the total genes, and the thinner lines the ${\bar{R}}_{r}$ values of the different kinetic classes of viral genes.

### Comparison of the effects on gene expression in three mutant PRV strains

**Figure 5:**
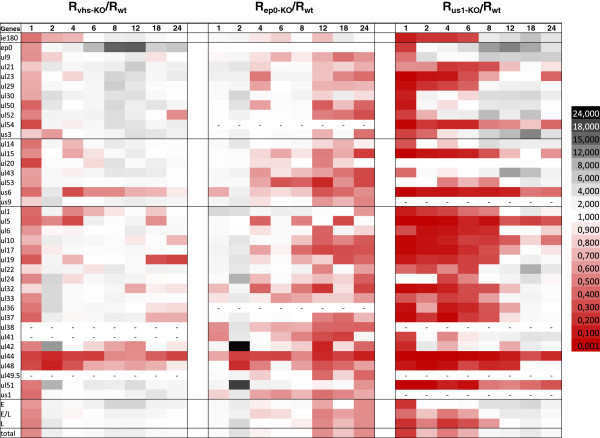
**Comparison of the gene expression patterns (the **${\bar{R}}_{r}$**values) in three mutant viruses.** The ${\bar{R}}_{r}$ values of the *us1*-, *ep0*- and *vhs*-null mutants were visualized in a heatmap presentation. The expression profile of the *us1-KO* virus is similar to that of the *vhs-KO*, i.e. complementary to that of the *ep0-KO* virus.

We compared the gene expression profiles (

$${\bar{R}}_{\;r}$$

values) of three mutant viruses: the *vhs-KO*[30], *ep0-KO*[28] and *us1-KO* viruses (Figure 5). On a broad scale, the *us1* and *vhs* gene deletions appeared to produce a similar overall expression pattern (though the *us1* deletion exerted a more marked effect), whereas *ep0* deletion led to an overall expression profile complementary to those of the *us1-KO* and *ep0-KO* viruses. Figure 5 reveals that the *us1* and *vhs* gene deletions resulted in down-regulation of the PRV genes in the E phase and in up-regulation of these genes in the L phase, whereas a reverse expression pattern was observed in the *ep0* mutant background. Furthermore, in both the *us1-KO* and the *vhs-KO* viruses, the most affected gene was the *ep0*, which was significantly up-regulated from 2 h pi. These results suggest a fundamental role of the EP0 transactivator in the formation of the global gene expression profile of PRV.

### The expression of the *ie180* gene is correlated with the expressions of the PRV genes in the *us1-KO* virus

We previously reported that the expression of the *ie180* gene, the major transactivator of the *wt* virus, was uncorrelated with the expressions of the remaining genes [30]. When we investigated this relationship in the *us1-KO* virus by using Pearson correlation analysis, very high correlations emerged between the *ie180* transcripts and the transcripts produced by all three kinetic classes of the PRV genes (Additional file 5A). The correlation was especially high between the *ie180* and the E transcripts. Interestingly, we observed a similar effect in the *vhs*-null mutant as concerns of the correlation between the *ie180* and the other viral genes [30].

### The initiation of DNA replication is delayed in the *us1-KO* as compared with the *wt* virus

**Figure 6:**
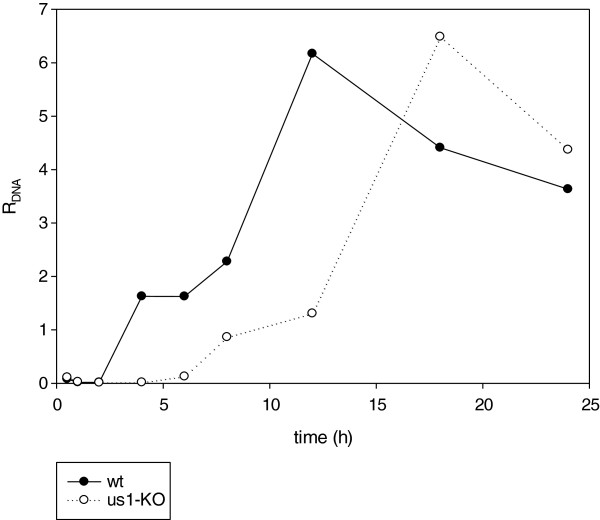
**Synthesis of viral DNAs.** The replication of viral DNA was monitored in the *wt* and the *us1-KO* viruses through the use of real-time RT-PCR. A 4-h delay was observed in the onset of DNA replication.

Our real-time RT-PCR investigation of the kinetics of DNA replication of the two viral strains demonstrated that the amount of viral DNA was slightly less at 1 h pi than at 30 min pi in both viruses (Figure 6). We can only speculate about this phenomenon. It is possible that the cellular DNases digest some proportion of the infecting viral DNA molecules. DNA synthesis was initiated between 2 and 4 h pi in the *wt* virus. There was a decrease in the rate of multiplication of the DNA within the interval 4–6 h pi, which might be due to the switch between the theta-type and rolling circle-type of replications. A high rate of amplification of DNA molecules was observed between 6 and 12 h pi, followed by a slow decrease in the copy number of the *wt* viral DNA. The decrease in the amount of viral DNA in the late stage of viral infection is explained by the egress of mature virions from the infected cells. The onset of the DNA replication of the mutant virus exhibited a significant delay relative to that of the *wt* virus. The *us1-KO* DNA started replication 4 h later, and the rate of production of viral genomes was lower than that of the *wt* virus within the period 6–12 h pi. The copy numbers of the viral genomes were almost exactly the same at their peaks in the two viruses, but the maximum amount was reached 6 h later in the mutant virus. The mechanism whereby ICP22 affects the DNA replication remains to be determined, but it may be associated with the effect of the *us1* mutation on the delay of the onset of the general gene expression. Since the expressions of several key viral factors, such as the IE180 and the EP0, were profoundly changed in consequence of the mutation, the effect of ICP22 on the DNA replication, at least in part, must be indirect.

### Investigation of the expression kinetics of PRV genes in the mutant and *wt* viruses through the use of R values normalized to the relative copy numbers of the DNA

**Figure 7:**
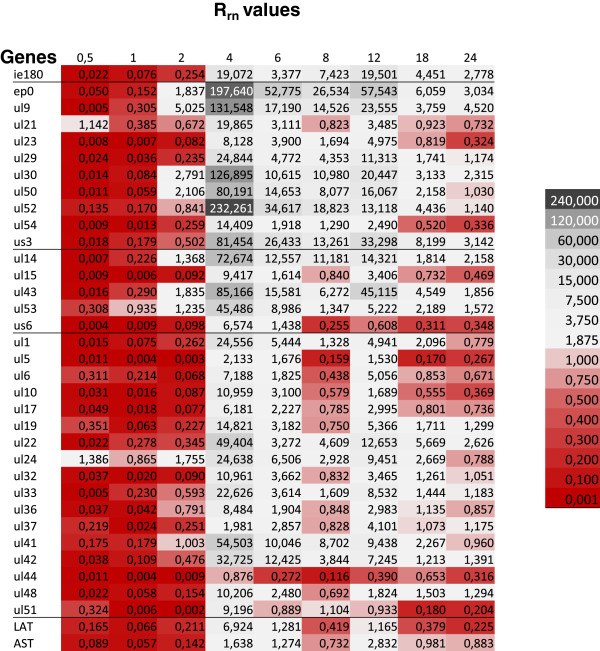
**Heatmap visualization of the ratios of the transcripts of the *us1-KO *and the *wt *viruses normalized to the relative copy number of the DNAs (R**_**rn**_ **= R**_**us1KO**_**:R**_**DNA-us1KO**_**/R**_**wt**_**:R**_**DNA-wt**_**).** The R_rn_ values were very low in the first 2 h pi. There was a drastic change in the period 4–6 h pi, when the R_rn_ values became high. In the last stage of infection, the gene expressions became the same in the two viruses.

**Figure 8:**
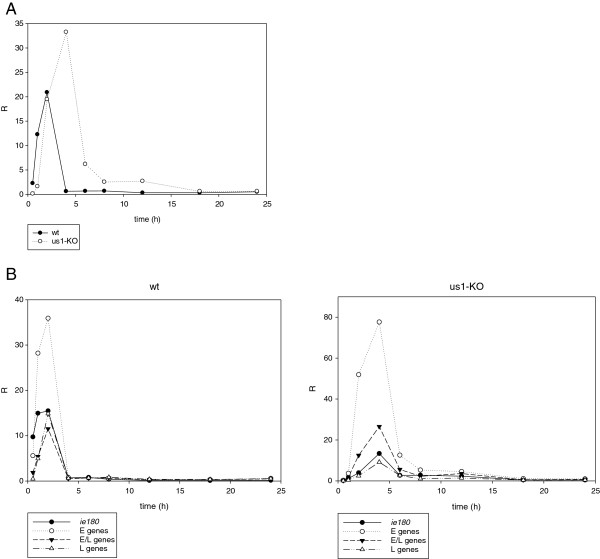
**The average transcription rates are drastically decreased after the onset of DNA replication.** The transcriptions from the genome of both genotypes are virtually shut down following the initiation of DNA synthesis. The transcription from the *us1-KO* genome is suppressed to a lower extent than that of *wt* virus.

We analyzed the viral gene expressions by normalizing the amounts of transcripts to the relative copy number of the DNA molecules, which allows a comparison of the transcriptional activity with the corresponding copy number of the DNA at different time points. The ratios of the normalized R values (R_rn_ = R_n-us1KO_/R_n-wt_) indicated that the level of transcripts in most genes was lower in the mutant than in the *wt* virus within the period 0.5-1 h pi with two exceptions (*ul21* and *ul24* at 30 min pi). This tendency was substantially reversed at 4 h pi, when the genes of the mutant virus were more active than those of the *wt* virus (Figure 7). Some early genes (*ep0*, *ul9*, *ul30*, and *ul52*) produced > 100 times more transcripts from a single copy of the genes in the mutant than in the *wt* virus. There was not such a large difference between the non-normalized genes of the two viruses. The switch from low to high amounts of transcripts occurred much later in the mutant virus, the exact time of the switch varying from gene to gene (Figure 2). The average expression from a single gene was very low at 30 min and 1 h pi in the *us1-KO* virus as compared with the *wt* virus, but it had become > 50 times higher (R_us1KO_/R_wt_) by 4 h pi (Figure 8A). This value was especially high for the average E genes (101.5), lower for the E/L genes (53), and much lower for the L genes (15.2). This phenomenon obviously resulted from the delayed onset of DNA synthesis in the *us1-KO* virus, which suggests that DNA replication exerts a severe constraint on the transcription. Indeed, the gene transcription rate was profoundly decreased after the initiation of DNA synthesis (Figures 8A and 8B). In the *wt* virus, the average rate of gene transcription was 33 fold less at 4 h pi than at 2 h pi (R_4h_/R_2h_), and remained low or even became lower. In the mutant virus, the decrease in the rate of transcription started later (from 4 h pi), and occurred gradually, not suddenly as in the *wt* virus. The steepest drop in gene expression in the interval 4–6 h pi did not coincide with the highest rate of DNA synthesis, at 6 to 8 h pi, which suggests that the inhibition of overall gene expression is not directly controlled by DNA replication itself, as it was suggested by Huvet and colleagues [31]. From data obtained in the analysis of human cells, those authors proposed novel gene expression regulatory mechanism, based on the collision of the transcriptional and DNA replication machineries. However, those results could not be reproduced by Necsulea and colleagues [32]. Although, it is generally conceived that the switch in expression from the E to the L kinetic class of genes is triggered by DNA replication, Figure 8B clearly shows that the maximum expression as regards the individual L genes occurs at 2 h pi, i.e. before the onset of DNA replication, which casts severe doubt on the validity of this idea.

### Normalization of the R values to the relative DNA copy number leads to the *ie180* gene correlating with the other viral genes in the *wt* background

Normalization of the R values of the cDNAs to the copy number of the viral DNAs leads to a very strong correlation between the *ie180* and the other genes in both genetic backgrounds (Additional file 5B). It is noteworthy that the strength of the correlation between the *ie180* and the different kinetic classes of genes is the opposite that in the non-normalized case. These data suggest that the synchronism in the transcription between the IE180 transactivator and the other genes is indicative of a real correlation. However, since the DNA replication imposes a severe constraint on the transcription of each gene, including the *ie180* gene, the high correlation between the normalized data could possibly be merely a statistical curiosity without any functional significance. Additional file 6 shows the R values of *ie180*, the average total viral genes and the average of various kinetic classes of genes normalized to the DNA copy number at different time points of infection.

## Conclusions

Our kinetic data show that the abrogation of the *us1* function leads to a significant reduction of transcription in every kinetic class of genes in the first hour pi. In the period 1 to 6 h pi, the L genes are selectively down-regulated, and the E genes are later up-regulated in the *us1-KO* background relative to the *wt* virus. The questions as to whether the ICP22 protein exerts a direct effect upon the gene expression and, if so, at what levels remains to be answered. It is noteworthy that deletion of the *vhs* gene resulted in a similar overall expression pattern as that observed in the *us1-KO* virus, and in an expression profile complementary to those in the *ep0-KO* virus. Interestingly, *ep0* was the gene that was most affected in both the *us1* and *vhs* knockout viruses (highly elevated expressions), which might imply that EP0 could be a common link in the determination of the overall gene expression profile. We observed a strong inhibition of transcription during DNA replication. The question arises of whether the process of DNA replication itself could exert this inhibitory effect. In the mutant virus, the main inhibition in gene expression occurs between 4 and 6 h pi, while DNA synthesis exhibits the highest rate 2 h later. This suggests that the decrease in gene expression is not, or not only, a result of the potential collision of the transcription and DNA replication machineries. We have earlier shown that disruption of the function of the *vhs* gene of the virus results in synchronization of the gene expression profile of the *ie180* and the remaining PRV genes [30]. We obtained very similar results with the *us1* gene-deleted PRV: the expression of the *ie180* gene correlated with those of the other PRV genes in the mutant virus, which was not the case for the *wt* virus. The question may be posed of whether this correlation is directly associated with the disruption of the *us1* gene function, or is rather caused by the delay of DNA replication.

## Methods

### Cells and viruses

Monolayer cultures of porcine kidney epithelial cells (PK-15) were used for the propagation of the pseudorabies virus. Cells were grown in DMEM (Sigma Aldrich), supplemented with 5% fetal bovine serum (Gibco) and 80 μg of gentamicin per ml (Invitrogen) at 37°C in the presence of 5% CO_2_. The Kaplan *wt* strain of PRV was used as the parental strain for the generation of the *us1*-null mutant virus (*us1-KO*).

### Construction of the *us1* gene knockout virus

The *Ka-us1-KO* (*us1-KO*, in short) virus was generated as follows. As a first step the *Bam*HI-10 fragment of PRV was isolated from the gel, and was then subcloned to pRL525 [33], resulting in the generation of pRL525-B10. This plasmid was used as a template for the PCR amplification of the two arms of the flanking sequences providing homology with the target viral genomic region. A green-fluorescent protein (GFP) gene expression cassette (Clontech) was inserted into the unique *Ecl136*II site of the targeting sequence, resulting in pUS1-gfp, which was used as the transfer plasmid for the generation of the knockout virus. The linearized transfer plasmid was transfected along with the purified *wt* viral DNA into PK-15 cells. The recombinant virus was generated by homologous recombination, then isolated and plaque-purified on the basis of the fluorescence. Rescued viruses were generated by using pUS1 as a transfer plasmid, which was co-transfected with the purified DNA of *us1-KO* to PK-15 cells. The revertant viruses were selected on the basis of the non-fluorescent plaque phenotype. Both mutant and rescued viruses were checked with DNA sequencing.

### Infection

The virus stock used for the experiments was prepared by infecting PK-15 cells with low-dose viruses, followed by incubation of the cells until a complete cytopathic effect was observed. To assess the effect of the *us1* gene deletion on the transcription kinetics of PRV, rapidly-growing semi-confluent PK-15 cells were infected with the *wt* or *us1-KO* virus at a high multiplicity of infection [MOI; 10 plaque forming units (pfu)/cell], and incubated for 1 h, which was followed by removal of the virus suspension and washing of the cells with PBS. Subsequently, fresh culture medium was added to the cells, which were further cultivated for an additional 0.5, 1, 2, 4, 6, 8, 12, 18 or 24 h.

### Isolation of RNAs

Infected or non-infected PK-15 cells were washed with PBS and harvested for RNA purification. Total RNA was isolated by using the NucleoSpin RNA II Kit (Macherey-Nagel GmbH and Co. KG) as recommended by the supplier. Briefly, harvested cells were collected by low-speed centrifugation, and lysed in the buffer included in the kit. Samples were treated with RNase-free rDNase solution (included in the Kit) to remove potential genomic DNA contamination. As the next step, the potential residual DNA contamination was removed by using Turbo DNase (Ambion Inc.). Subsequently, RNA samples were eluted in RNase-free water (supplied with the kit), resulting in a total volume of 60 μl of RNA solution. RNA concentrations were measured spectrophotometrically in a BioPhotometer Plus (Eppendorf). The RNA solution was stored at −80°C until use.

### Reverse transcription

Total RNA samples were reverse-transcribed by using gene-specific primers, and SuperScript III reverse transcriptase (Invitrogen) as described in our earlier reports [9,34]. Briefly, RT mixtures containing total RNA, primer, SuperScript III enzyme, buffer, dNTP mix and RNase inhibitor (RNAsin, Promega) were incubated at 55°C for 1 h. The amplification of the first-strand cDNA synthesis was terminated by keeping the samples at 70°C for 15 min. The cDNAs were diluted 10-fold with nuclease-free water (Promega Corp.) and the solutions were stored at −80°C until use.

### Real-time PCR

SYBR Green-based (Absolute QPCR SYBR Green Mix, Thermo Scientific) quantitative real-time PCRs were performed on the first-strand cDNAs in a real-time PCR cycler (Rotor-Gene 6000, Corbett Life Sciences), as described in our previous studies [9,30]. The specificity of the reverse transcription and the PCR reactions was ensured by using no-RT, no-primer, and no-template controls. The accuracy of sampling was guaranteed by using 28S rRNA of swine as loading control. The specificity of the PCR products was confirmed by melting point analysis, PAGE and/or DNA sequencing.

### DNA sequencing

We used pyrosequencing with a Pyromark Q24 pyrosequencer (Qiagen) to validate the mutation of the *us1* gene and the specificity of the PCR products obtained in the kinetic experiments in the event of doubt.

### Data analysis

The relative expression ratios (R values) of the cDNAs of the PRV genes were calculated via the following formula:

R=Esample6hCtsample6hEsampleCtsample¯:Eref6hCtref6hErefCtref¯

The cDNAs were all normalized to the cDNAs of the 28S rRNAs of swine [9] by using the Comparative Quantitation module of the Rotor-Gene 6000 software (Version 1.7.28, Corbett Research), which automatically sets the thresholds and calculates the efficiency of PCR reactions. We used the average 6-h ECt values of the “samples”, with those of the “references” as controls, as in our earlier works [9,30,34]. The R values of the viral DNAs were calculated similarly; the 6-h ECt values were taken as the control, and porcine 28S rRNA gene was used as the reference. The effect of deletion of the *us1* gene on the global gene expression was calculated by using R_r_, the ratio of the R values of the *us1* mutant and the *wt* viruses (R_r_ = R_us1KO_/R_wt_), where R_us1KO_ and R_wt_ are the R values of a particular gene at a given time point in the *us1*-KO and *wt* genetic background, respectively. All data were analyzed by using the average and the standard deviance functions of Microsoft Excel. Pearson's correlation coefficient was calculated for the analysis of the correlation between the expression kinetics of the genes, using the following formula:

$r=\frac{\sum_{i=1}^{n}\left(X_{i},-,\bar{X}\right)\left(Y_{i},-,\bar{Y}\right)}{\left(n-1\right)S_{x}S_{y}}$[35]. The normalized R values were calculated by dividing the appropriate R value of the cDNA by the R value of the viral DNA measured at the same time for the same sample. The Pearson correlation coefficient is a number between −1 and +1 that measures the linear relationship between two variables, denoted here as X and Y, which are the R values of two different genes or the average R values of genes belonging in the same kinetic class in the same time interval. X and Y are the average values, n is the sample number, and SX and SY are the standard errors of the mean values for X and Y, respectively. A positive value for the correlation indicates a positive association, while a negative value indicates an inverse association.

## Competing interests

The authors declare that they have no competing interests.

## Authors’ contributions

IFT carried out the construction of the targeting plasmids, RNA purification, the reverse transcription reactions, the standard and real-time PCR and. DT carried out the DNA sequencing, and participated in the evaluation of the primary data. BB took part by performing the RT reactions and the real-time PCR. IP purified PRV RNA, propagated PK-15 cells and participated in the genotyping of the recombinant virus. NP carried out the agarose and polyacrylamide gel electrophoresis and participated in the propagation of cultured cells. ZB coordinated the study, propagated viruses, isolated the viral DNAs and isolated the recombinant viruses. All authors have read and approved the final manuscript.

## Supplementary Material

Additional file 1 **Comparison of the rates of increase of viral DNA during the first twelve hours of PRV infection.** We observed similar dynamics in the growth rates of the DNA of the wild-type and rescued PRVs, while both differed significantly from those of the us1-mutant virus. Click here for file

Additional file 2 **Comparison of the transcription kinetics of the *ie180 *gene in the wild-type, *us1*-KO and *us1*-rescued PRV.** This revealed that the kinetics of the rescued virus resembled that of the wild-type PRV, but differed significantly from that of the mutant virus. Click here for file

Additional file 3 **Comparison of the transcription kinetics of the *ep0 *gene in the wild-type, *us1*-KO and *us1*-rescued PRV.** This revealed that the kinetics of the rescued virus resembled that of the wild-type PRV, but differed significantly from that of the mutant virus. Click here for file

Additional file 4 **The average relative expression ratios (**$\bar{R}$**).** This table shows the $\bar{R}$ values for the total PRV genes (A) and for each kinetic class of viral genes (B) at different time points of infection. Click here for file

Additional file 5 **Correlations between the transcription of the *ie180 *gene and other PRV genes in *wt * and *us1-KO * backgrounds A.** The viral genes are expressed in synchronism with the *ie180* gene in the mutant virus, whereas the expressions are not correlated in the *wt* virus. B. Correlation between the transcription of the *ie180* gene and other PRV genes in the *wt* and *us1-KO* backgrounds with the use of normalized R values. The expression of viral genes becomes correlated with the expression of *ie180* genes in the *wt* virus, too. Click here for file

Additional file 6 **The normalized average relative expression ratios (**${\bar{R}}_{n}$**) values This table shows the**${\bar{R}}_{n}$** values for the total PRV genes (A) and for each kinetic class of viral genes (B) at different time points of infection.** Click here for file
