# Supplementary figures and images for: The ICP22 protein selectively modifies the transcription of different kinetic classes of pseudorabies virus genes
Source: BMC Mol Biol. 2013 Jan 29;14:2. doi: 10.1186/1471-2199-14-2 (PMC3599583; doi:10.1186/1471-2199-14-2)

## Slide 1
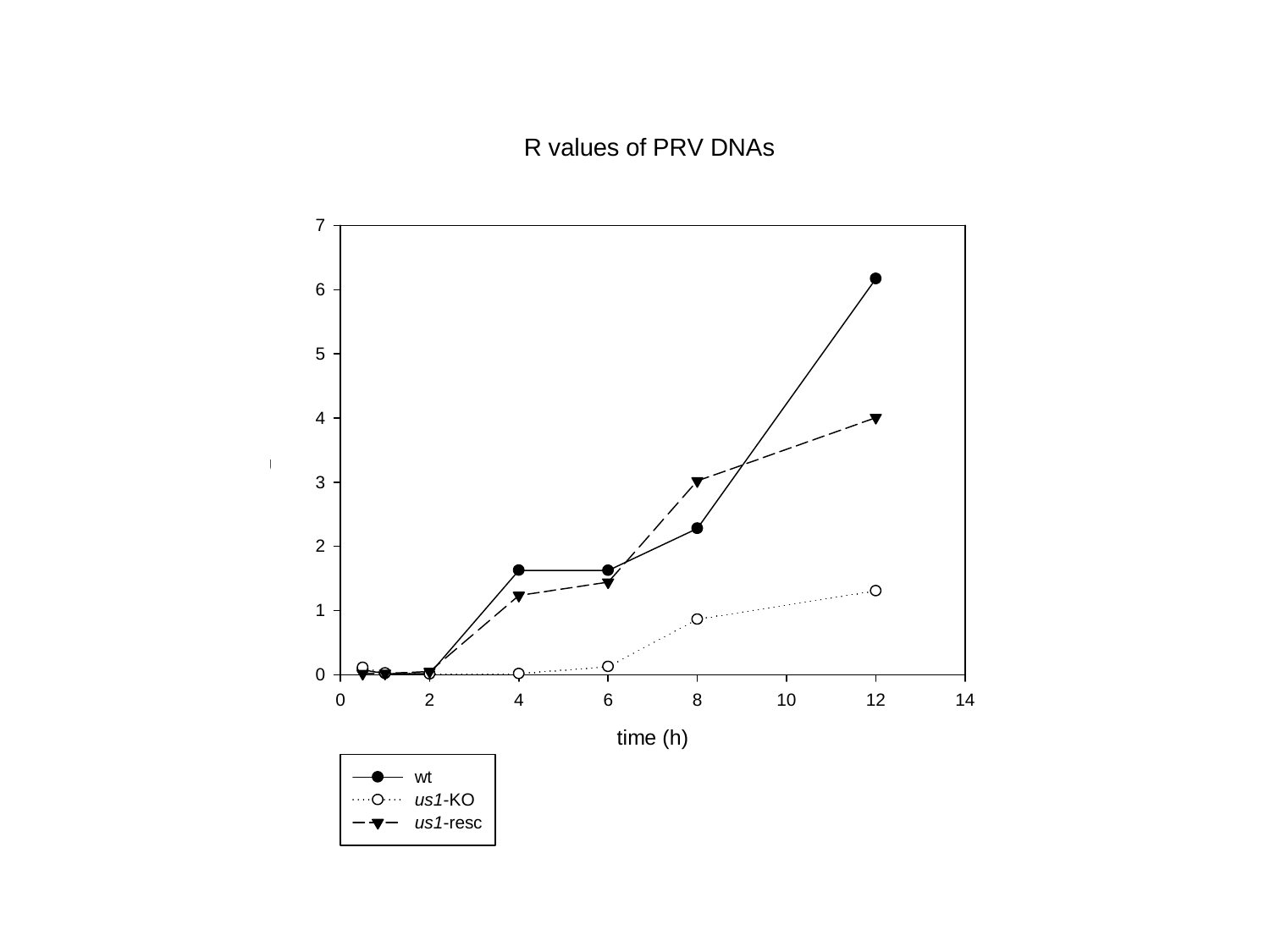

Supplement: Additional file 1 — Comparison of the rates of increase of viral DNA during the first twelve hours of PRV infection. We observed similar dynamics in the growth rates of the DNA of the wild-type and rescued PRVs, while both differed significantly from those of the us1-mutant virus. [file 1471-2199-14-2-S1.ppt]

## Slide 1
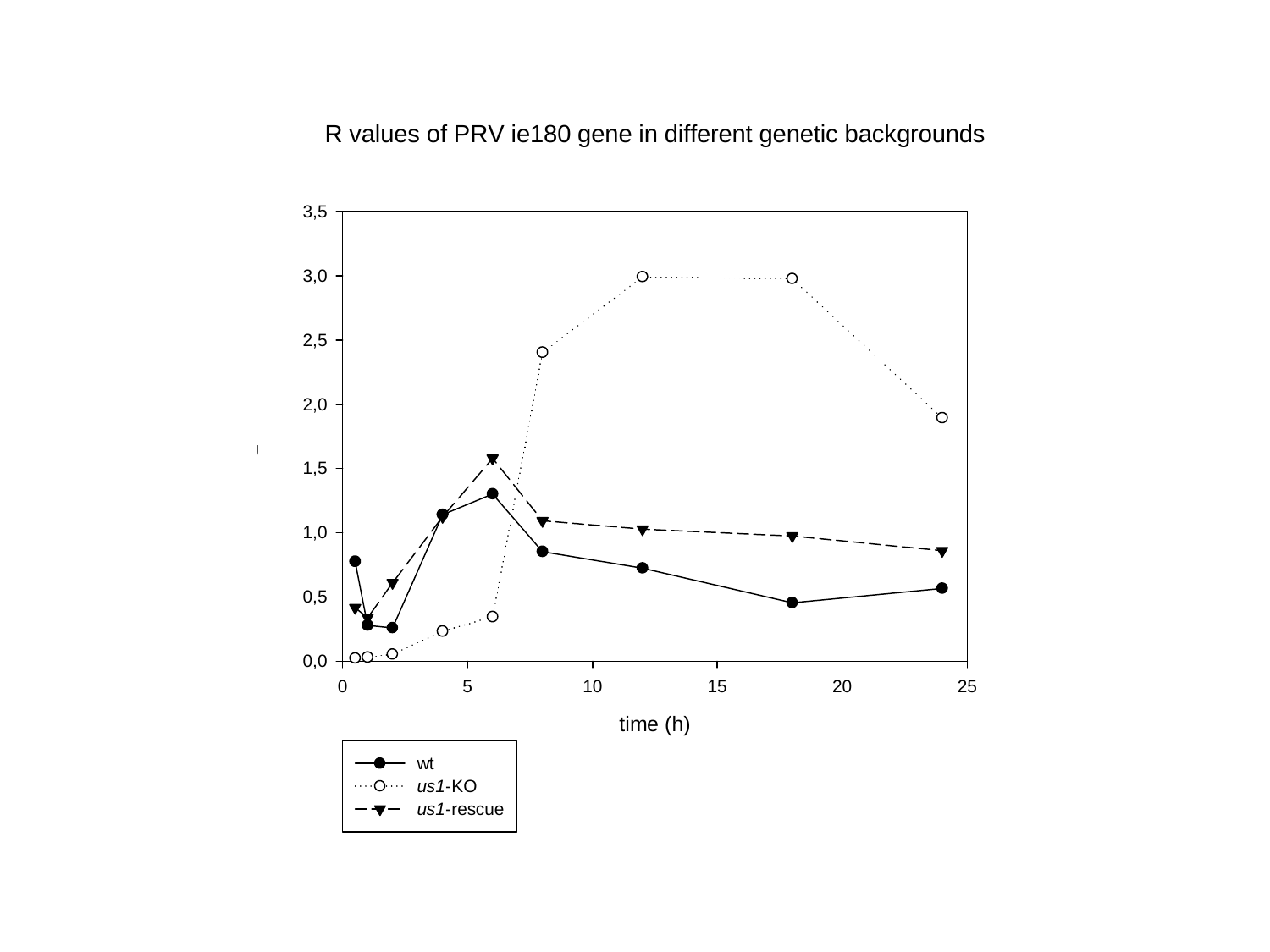

Supplement: Additional file 2 — Comparison of the transcription kinetics of the ie180 gene in the wild-type, us1-KO and us1-rescued PRV. This revealed that the kinetics of the rescued virus resembled that of the wild-type PRV, but differed significantly from that of the mutant virus. [file 1471-2199-14-2-S2.ppt]

## Slide 1
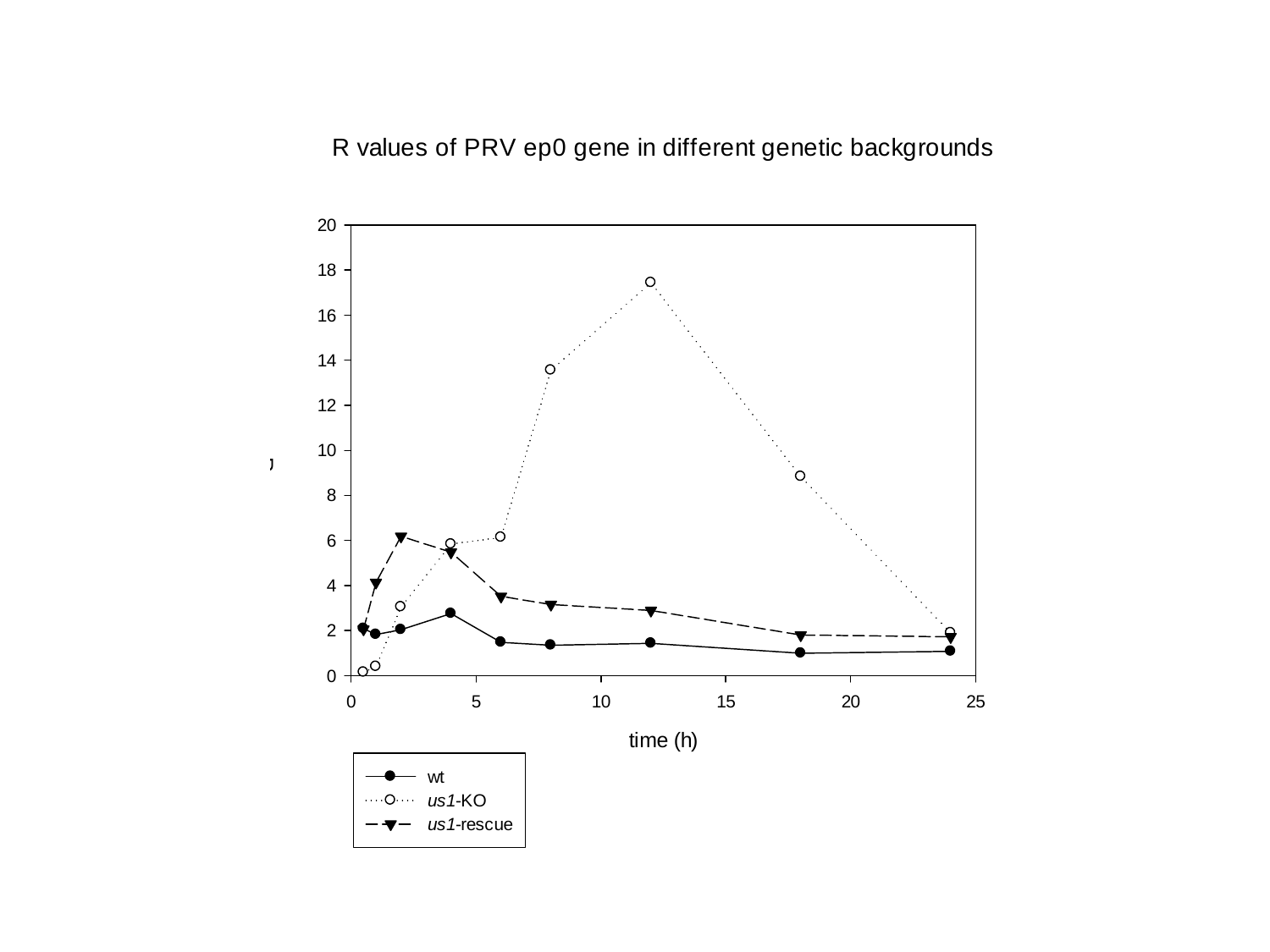

Supplement: Additional file 3 — Comparison of the transcription kinetics of the ep0 gene in the wild-type, us1-KO and us1-rescued PRV. This revealed that the kinetics of the rescued virus resembled that of the wild-type PRV, but differed significantly from that of the mutant virus. [file 1471-2199-14-2-S3.ppt]
