# Supplementary material for: The ICP22 protein selectively modifies the transcription of different kinetic classes of pseudorabies virus genes
Source: BMC Mol Biol. 2013 Jan 29;14:2. doi: 10.1186/1471-2199-14-2 (PMC3599583; doi:10.1186/1471-2199-14-2)
Supplement: Additional file 4 — The average relative expression ratios (R¯). This table shows the R¯ values for the total PRV genes (A) and for each kinetic class of viral genes (B) at different time points of infection. [file 1471-2199-14-2-S4.docx]

**Table 1.** **The average relative expression ratios (**$\overline{R}$**)**

**A. Total genes**

| time (h) | 0.5 | 1 | 2 | 4 | 6 | 8 | 12 | 18 | 24 |
| --- | --- | --- | --- | --- | --- | --- | --- | --- | --- |
| ***wt*** | 0.182 | 0.229 | 0.351 | 1.036 | 1.111 | 1.600 | 2.037 | 1.465 | 1.914 |
| ***us1-KO*** | 0.017 | 0.044 | 0.266 | 0.580 | 0.793 | 2.220 | 3.569 | 4.176 | 2.846 |

**B. Kinetic classes of genes**

***wt***

| time (h) | 0.5 | 1 | 2 | 4 | 6 | 8 | 12 | 18 | 24 |
| --- | --- | --- | --- | --- | --- | --- | --- | --- | --- |
| ***ie180*** | 0.776 | 0.279 | 0.259 | 1.140 | 1.302 | 0.853 | 0.725 | 0.455 | 0.567 |
| **E** | 0.446 | 0.527 | 0.602 | 1.244 | 1.099 | 1.120 | 1.546 | 1.425 | 2.107 |
| **E/L** | 0.149 | 0.101 | 0.193 | 0.815 | 1.085 | 1.550 | 1.771 | 1.306 | 1.684 |
| **L** | 0.038 | 0.092 | 0.249 | 0.978 | 1.126 | 1.896 | 2.404 | 1.536 | 1.868 |
| ***us1-KO*** |  |  |  |  |  |  |  |  |  |
| time (h) | 0.5 | 1 | 2 | 4 | 6 | 8 | 12 | 18 | 24 |
| ***ie180*** | 0.023 | 0.030 | 0.054 | 0.233 | 0.346 | 2.404 | 2.993 | 2.978 | 1.894 |
| **E** | 0.033 | 0.099 | 0.710 | 1.353 | 1.604 | 4.590 | 5.952 | 7.100 | 4.284 |
| **E/L** | 0.018 | 0.050 | 0.171 | 0.463 | 0.714 | 1.943 | 4.656 | 3.736 | 3.174 |
| **L** | 0.007 | 0.011 | 0.034 | 0.159 | 0.339 | 0.907 | 1.848 | 2.585 | 1.903 |
