# Supplementary material for: The ICP22 protein selectively modifies the transcription of different kinetic classes of pseudorabies virus genes
Source: BMC Mol Biol. 2013 Jan 29;14:2. doi: 10.1186/1471-2199-14-2 (PMC3599583; doi:10.1186/1471-2199-14-2)
Supplement: Additional file 5 — Correlations between the transcription of the ie180 gene and other PRV genes in wt and us1-KO backgrounds A. The viral genes are expressed in synchronism with the ie180 gene in the mutant virus, whereas the expressions are not correlated in the wt virus. B. Correlation between the transcription of the ie180 gene and other PRV genes in the wt and us1-KO backgrounds with the use of normalized R values. The expression of viral genes becomes correlated with the expression of ie180 genes in the wt virus, too. [file 1471-2199-14-2-S5.docx]

**Table 2.** Correlation between the transcripts of ie180 and the rest of the viral genes

**A. Gene expressions**

***wt us1-KO***

*ie180*/total 0.246 0.971

*ie180*/E 0.175 0.983

*ie180*/(E/L) 0.297 0.949

*ie180*/L 0.250 0.918

**B. Normalized gene expressions**

***wt us1-KO***

*ie180*/total 0.888 0.940

*ie180*/E 0.931 0.911

*ie180*/(E/L) 0.869 0.971

*ie180*/L 0.769 0.988
