# Supplementary material for: The ICP22 protein selectively modifies the transcription of different kinetic classes of pseudorabies virus genes
Source: BMC Mol Biol. 2013 Jan 29;14:2. doi: 10.1186/1471-2199-14-2 (PMC3599583; doi:10.1186/1471-2199-14-2)
Supplement: Additional file 6 — The normalized average relative expression ratios (R¯n) values This table shows theR¯n values for the total PRV genes (A) and for each kinetic class of viral genes (B) at different time points of infection. [file 1471-2199-14-2-S6.docx]

**Table 3.** The normalized R (R_n_) values

**A. Total genes**

***wt***

| time (h) | 0.5 | | 1 | | 2 | | 4 | | 6 | | 8 | | 12 | | 18 | | | 24 | |  |  |  |  |  |  |  |  |  |  |
| --- | --- | --- | --- | --- | --- | --- | --- | --- | --- | --- | --- | --- | --- | --- | --- | --- | --- | --- | --- | --- | --- | --- | --- | --- | --- | --- | --- | --- | --- |
| *ie180* | 9.730 | | 14.956 | | 15.471 | | 0.701 | | 0.801 | | 0.374 | | 0.117 | | 0.103 | | | 0.156 | |  |  |  |  |  |  |  |  |  |  |
| total | 2.289 | | 12.283 | | 20.915 | | 0.637 | | 0.684 | | 0.702 | | 0.330 | | 0.332 | | | 0.527 | |  |  |  |  |  |  |  |  |  |  |
| ***us1-KO*** | |  | |  | |  | |  | |  | |  | |  | |  |  | |  |  |  |  |  |  |  |  |  |  |  |
| time (h) | | 0.5 | | 1 | | 2 | | 4 | | 6 | | 8 | | 12 | | 18 | 24 | |  |  |  |  |  |  |  |  |  |  |  |
| *ie180* | | 0.211 | | 1.129 | | 3.931 | | 13.368 | | 2.705 | | 2.779 | | 2.291 | | 0.459 | 0.433 | |  |  |  |  |  |  |  |  |  |  |  |
| total | | 0.150 | | 1.647 | | 19.490 | | 33.269 | | 6.204 | | 2.566 | | 2.732 | | 0.644 | 0.651 | |  |  |  |  |  |  |  |  |  |  |  |
|  |  | |  | |  | |  | |  | |  | |  | |  | | |  | |  |  |  |  |  |  |  |  |  |  |

**B. Kinetic classes of genes**

***wt***

| time (h) | 0.5 | 1 | 2 | 4 | 6 | 8 | 12 | 18 | 24 |
| --- | --- | --- | --- | --- | --- | --- | --- | --- | --- |
| *ie180* | 9.730 | 14.956 | 15.471 | 0.701 | 0.801 | 0.374 | 0.117 | 0.103 | 0.156 |
| E | 5.587 | 28.214 | 35.913 | 0.765 | 0.676 | 0.492 | 0.251 | 0.323 | 0.580 |
| E/L | 1.874 | 5.400 | 11.524 | 0.501 | 0.668 | 0.681 | 0.287 | 0.296 | 0.463 |
| L | 0.470 | 4.937 | 14.855 | 0.601 | 0.693 | 0.832 | 0.390 | 0.348 | 0.514 |
| ***us1-KO*** |  |  |  |  |  |  |  |  |  |
| time (h) | 0.5 | 1 | 2 | 4 | 6 | 8 | 12 | 18 | 24 |
| *ie180* | 0.211 | 1.129 | 3.931 | 13.368 | 2.705 | 2.779 | 2.291 | 0.459 | 0.433 |
| E | 0.295 | 3.664 | 51.932 | 77.630 | 12.552 | 5.305 | 4.557 | 1.095 | 0.980 |
| E/L | 0.162 | 1.843 | 12.498 | 26.570 | 5.587 | 2.246 | 3.564 | 0.576 | 0.726 |
| L | 0.061 | 0.403 | 2.464 | 9.145 | 2.651 | 1.048 | 1.414 | 0.398 | 0.435 |
